# 3D reconstruction of a gallbladder duplication to guide LC: A case report and literature review

**DOI:** 10.1097/MD.0000000000033054

**Published:** 2023-02-22

**Authors:** Yong Qing Ye, Qing Liang, En Ze Li, Jing Lin Gong, Jing Ming Fan, Ping Wang

**Affiliations:** a Department of Hepatobiliary Surgery, The First Affiliated Hospital of Guangzhou Medical University, Guangdong Province, Guangzhou, China.

**Keywords:** gallbladder duplication, laparoscopic cholecystectomy, surgery, 3-dimensional reconstruction, treatment

## Abstract

**Rationale:**

Gallbladder duplication is a congenital aberration of the biliary tree, which is rarely encountered in the abdomen. It is a challenge that can be encountered by surgeons and is associated with an increased risk of complications after cholecystectomy. More than 50% of gallbladder duplication cases were undetected on preoperative traditional imaging. In this study, a case of gallbladder duplication in a patient with mild abdominal pain detected using preoperative 3-dimensional (3D) reconstruction of the gallbladder was described for the first time.

**Patient concerns and diagnosis:**

We present a case of gallbladder duplication in a 32-year-old man who was referred to our hospital for recurrent right upper quadrant abdominal pain without any other significant history.

**Interventions and outcomes:**

He underwent a 3D reconstruction technique as a supplement for gallbladder duplication that could not be diagnosed using magnetic resonance cholangiopancreatography or other traditional tools. Compared with other diagnostic tools, 3D reconstruction is more visual and accurate for diagnosing gallbladder duplication and guiding laparoscopic cholecystectomy without ductal injuries or other complications.

**Conclusion:**

Gallbladder duplication is an extremely rare biliary anatomical anomaly; failure to recognize it perioperatively exposes the patient to an increased risk of bile duct injuries. We review 28 cases of missed gallbladder duplication and conclude that less 50% of gallbladder duplication cases were detected via preoperative traditional imaging. We present a case and find that the 3D reconstruction technique can be used as a supplement for gallbladder duplication that could not be diagnosed by using magnetic resonance cholangiopancreatography or other tools. The value of using 3D reconstruction of gallbladder duplication is feasible and innovative, and facilitates guiding to laparoscopic cholecystectomy.

## 1. Introduction

Gallbladder duplication is an uncommon congenital anomaly, which is rarely detected by ultrasonography preoperatively. A few diseases, like hepatic cysts and choledochal cysts, can mimic a gallbladder duplication, thus increasing the risk of problematic intervention and unnecessary open surgery. A 3-dimensional (3D) reconstruction is a modern computer-aided surgical technique that converts CT or MRI data into a 3D model using procedural imaging operations. Recently, 3D reconstruction has developed rapidly, enabling precise calculation of total liver volume and vascular system. Additionally, it helps in the clear visualization of the anatomy of the biliary system.^[1]^ However, the application of 3D reconstruction in abnormal anatomy of the gallbladder to guide laparoscopic cholecystectomy (LC) has not yet been reported. In this study, a case of the gallbladder duplication in a patient with mild abdominal pain detected by preoperative 3D reconstruction of the gallbladder is described for the first time.

## 2. Case presentation

**Figure 1.**
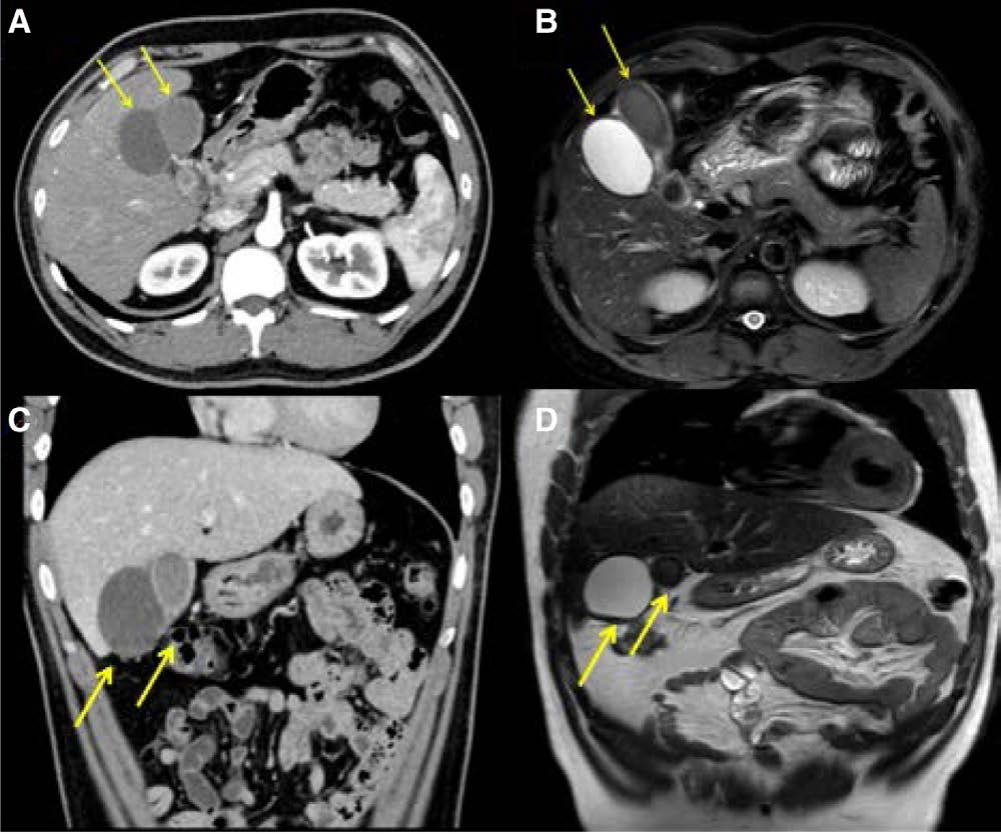
Computed tomography images and magnetic resonance imaging of the double gallbladders. (A) Abdominal CT revealed a 53 × 40 mm low density on the right gallbladder and a high-density shadow with an enhancing thick cystic wall on the left side of the gallbladder. (B) Abdominal MRI revealed a high possibility of a stone-packed double gallbladder but failed to visualize the cystic duct. (C) The coronal section of CT image of gallbladder (D) The coronal section of MRI image of gallbladder.

A 32-year-old man was referred to our hospital for recurrent right upper quadrant abdominal pain without any other significant history. His laboratory details like liver function test, CBC, and other inflammatory markers were negative. His initial abdominal B-scan revealed 2 cystic masses, 53 × 45 mm on the right and 68 × 27 mm on the left, both filled with massive stones. The common bile duct was normal in caliber. A follow-up abdominal CT and MRI revealed that cholestasis was present in the left cystic cavity with chronic cholecystitis, and stones were seen in the right cystic cavity. The cystic duct could not be observed (Fig. 1). Magnetic resonance cholangiopancreatography (MRCP) has an added advantage in revealing anomalous anatomy. However, due to severe gallbladder cholestasis, MRCP could not demonstrate the shape and location of the gallbladder and cystic duct.

**Figure 2.**
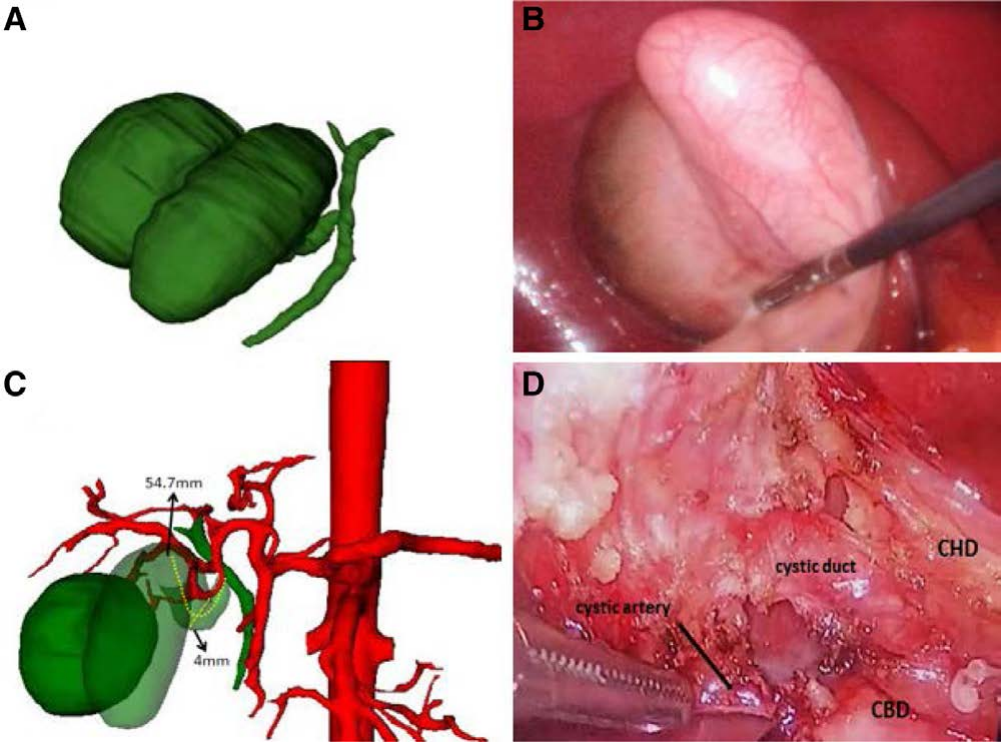
Compared 3D reconstruction with a laparoscopic photograph of the double gallbladders. (A) 3D reconstruction of double gallbladders. (B) 3D reconstruction of the spatial position of the double gallbladder and cystic artery. (C) Laparoscopic photograph of double gallbladders. (D) Laparoscopic photograph of the 2 separate gallbladders with a single cystic duct and a cystic artery.

**Figure 3.**
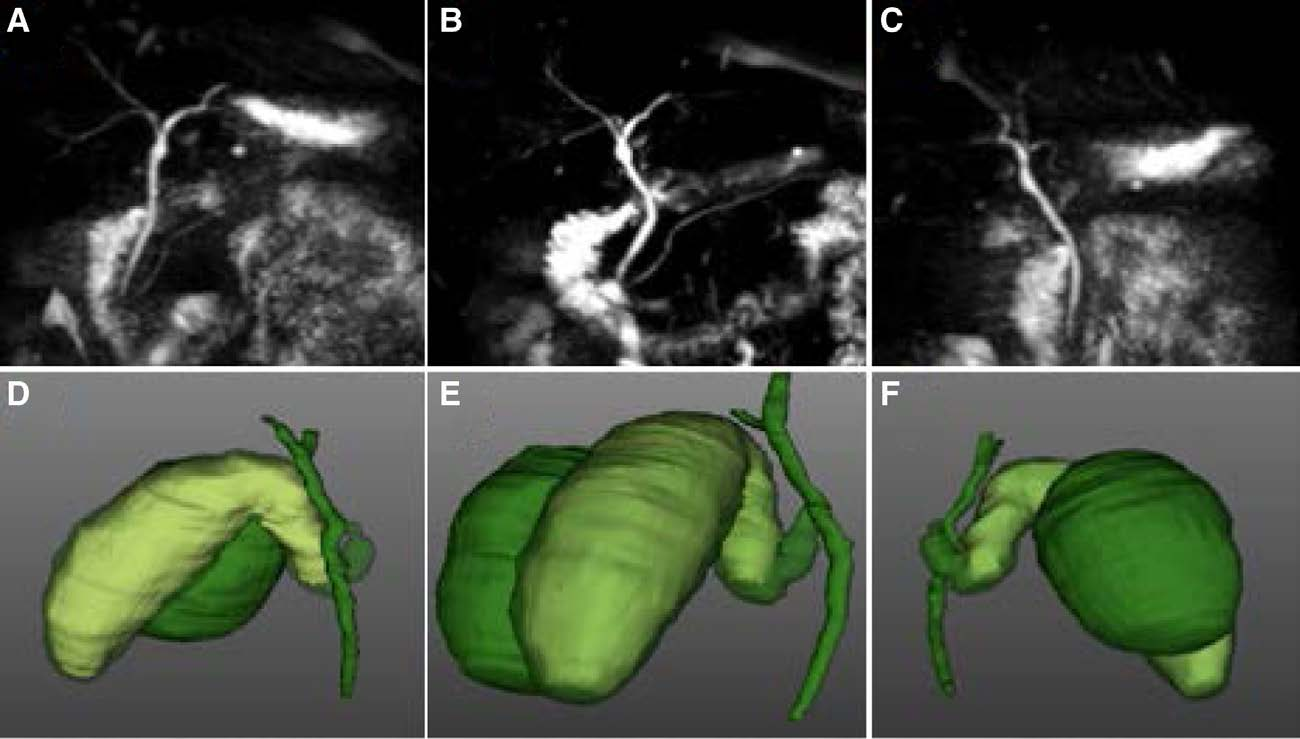
Compared 3D reconstruction with magnetic resonance cholangiopancreatography of the double gallbladders. Presentation of different perspectives between 3D reconstruction and MRCP. The double gallbladders cannot be imaged by MRCP.

A 3D reconstruction of the gallbladder was recommended to investigate the anomalous anatomy of the patient. The gallbladder duplication were simulated using the 3D CT software (IQQA-3D, China) based on preoperative dynamic helical CT. The 3D reconstruction technology was used to simulate the spatial relationship of the double gallbladder, cystic duct, and cystic artery of the patient (Fig. 2). Compared to MRCP, 3D reconstruction revealed that the gallbladder duplications were adjacent to each other with a single cystic duct, presenting a V-shaped pattern (Fig. 3). The predicted length and width of the cystic duct were 57.4 mm and 4 mm, respectively, which fitted the 5 mm hemlock. The position of Calot triangle was also seen (Fig. 2).

**Figure 4.**
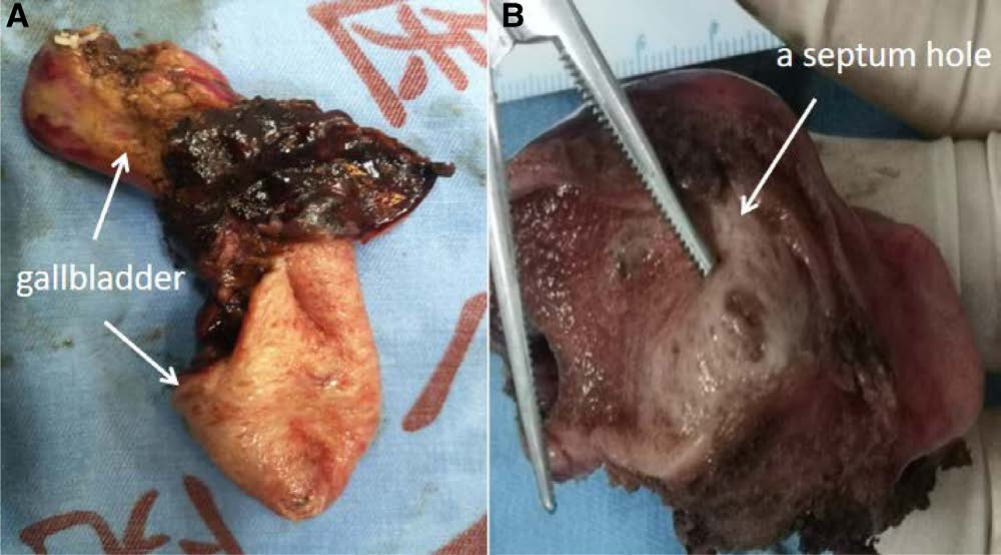
Resected specimen. (A) It presented a V-shaped pattern. (B) The gross specimen revealed a septum hole in 2 halves.

The patient was scheduled for laparoscopic cholecystectomy the next day. It was confirmed that the double gallbladder structure and position of the Calot triangle were consistent with the results of 3D construction at laparoscopic exploration. No cystic artery variants were identified. Finally, cholecystectomy was performed with accuracy and without any complications. The gross specimen examination of cholecystectomy revealed a septum hole in the middle dividing the gallbladder cavity into 2 halves, presenting a V-shaped pattern (Fig. 4). Further, histopathological examination of both the gallbladders revealed chronic cholecystitis. Also, one of the cystic cavities was completely organized due to long-term inflammation, resulting in no bile.

## 3. Discussion and conclusion

Gallbladder duplication, a rare congenital anomaly of the extrahepatic biliary system, is estimated at 1/4000 birth.^[2]^ It occurs due to the degradation of the caudal bud of the hepatic diverticulum leading to a split or bifurcate gallbladder, with earlier bifurcations causing a more complete duplication.^[3]^ It is difficult to detect this anomaly unless it has gallstones or cholecystitis resulting in any clinical significance. Boyden was the first 1 to describe gallbladder duplication and its classification in 1926. According to Boyden classification,^[4]^ it can be divided into vesical fellea divisa (double gallbladder with a common neck) and vesical fellea duplex (double gallbladders with separate cystic ducts). Harlaftis classifies double gallbladders into types 1 (split primordial) and 2 (accessory gallbladder).^[5]^ Type 1 has a single cystic duct. It is subdivided into septate, V-shaped, or Y-shaped. Type 2 has 2 or more cystic ducts, which are subdivided into H (or ductular) gallbladder and trabecular gallbladder. In our case, we presented a V-shaped gallbladder duplication which had occured in only 9.5% of cases.

The main imaging methods for gallbaldder duplication include ultrasound, CT, MR, and endoscopies retrograde cholangiography imaging. Although ultrasound is the effective tool for diagnosis of gallbladder duplication with stones, it is not effective in differentiating bilobed gallbladder from a true duplication.^[6]^ Abdominal CT clearly show gallstones, spatial location of gallbladder, and hepatic arteries and portal veins, but is not able to define biliary tree. MRCP imaging is the preferred method for delineating the anatomy of the biliary duct.^[7]^ MRCP has an accuracy of 95% in the diagnosis of cystic duct variants.^[8]^ However, MRCP images are obtained using the maximum intensity projection technique, which is a 2-dimensional imaging method. Additionally, MRCP could lead to an inaccurate diagnosis due to overlapping variant anatomy, torsion, or adhesion of the ducts, like in this case. The gallbladder was completely organized because of long-term chronic inflammation, resulting in no bile residue. Hence, imaging with MRCP was not possible in this case. Duplication of the gallbladder has been detected by oral cholecystography, scintigraphy, and endoscopies retrograde cholangiography, but these examinations are invasive imaging modalities.^[9]^ The 3D reconstruction technique is a new method that converts 2D CT images into 3D models using interactive imaging processing. Compared with CT, and MRCP images, 3D visualization and 3D models are more intuitive and more accurate in displaying the anatomical and spatial structure of the organs and have achieved great success.^[10]^ Vezakis^[11]^ used 3D MR cholangiopancreatogram clearly showing 2 separate cystic ducts. In our study, the 3D reconstruction models can be amplified, rotated, and hyalinized to clarify the anatomic character of the double gallbladders and their relationship with the surrounding bile ducts and blood vessels with omnidirectional, multiple-angle, and multilevel views. The 3D reconstruction of the gallbladder can provide anatomical details during surgery and avoid intraoperative damage to the biliary duct.^[12]^

For treatment of gallbladder duplication, most surgeons advocates LC becoming the treatment of choice for gallbladder duplication.^[13,14]^ However, as it was not easy to identify the anatomical structures safely, sometimes the procedure was converted to open cholecystectomy. Acutally, the majority of gallbladder duplication are missed in the preoperative diagnosis. Fifty presents of cases were undetected via preoperative imaging. Twenty-seven cases of missed gallbladder duplication that have been reported are presented in Table 1. Surgeons should be aware of this missed anomaly during the patient’s preoperative evolution and surgery as congenital anomalies are considered critical predisposing factors for bile duct injuries during cholecystectomy.

**Table 1:** Cases of missed gallbladder duplication.

First author	Year	Cases	Imaging method for missed diagnoses	Intraoperative or postoperative finding	Operation	Surgical complications
Garcia J^[15]^	1993	M, 21y	US/gastroduodenal endoscopy	intraoperative	LC	N
Cummiskey^[16]^	1997	F, 39y		intraoperative	LC	N
Yoganci^[17]^	2001	F, 48y	US/CT	intraoperative	LC	N
Weibel^[18]^	2001	-	US/cholangiographic/laparoscopy	postoperative	LC	conversion to OC
Papaziogas^[19]^	2005	F, 45y	US	intraoperative	LC	conversion to OC
Vijayaraghavan^[20]^	2006	M, 32y	US	intraoperative	LC	conversion to OC
Lefemire^[21]^	2009	M, 55y	laparoscopy	postoperative	LC	N
Desolneux G^[22]^	2009	M, 61y	US/MRCP	intraoperative	LC	bile duct stone and abscess
Causey M^[23]^	2010	F, 15y	US	intraoperative	LC	N
Guajardo–Salinas^[24]^	2010	M, 21y	US	intraoperative	LC	N
Walbolt and Lalezarzadeh^[25]^	2011	F, 36y	US/CT/MRCP	intraoperative	LC	N
Alam MT^[26]^	2011	F, 17y	US	intraoperative	LC	N
Nguyen VX^[27]^	2011	M, 73y	CT	postoperative	OC	N
Mulholland^[28]^	2012	-	US	intraoperative	LC	N
Ozaki N^[29]^	2014	F, 79y	US/CT/MRCP/ERCP/PET-CT	intraoperative	OC	N
Lo DD^[30]^	2015	F, 9month	US/MRCP	intraoperative	LC	N
Wong C^[31]^	2018	F, 23y	US/CT/MRCP	intraoperative	OC	N
Kumar M^[32]^	2018	F, 45y	US/MRCP	intraoperative	LC	N
Kowalchuk RO^[33]^	2018	M, 49y	US/CT/laparoscopy	postoperative	OC	N
Painuly^[34]^	2018	F, 61y	US	intraoperative	LC	N
Pera SJ^[35]^	2019	F, 45y	laparoscopy	postoperative	LC	N
Jia Z^[36]^	2020	F, 45y	US	intraoperative	EMIC	N
Alsharedah^[37]^	2020	F, 54y	US	intraoperative	LC	N
Zhuang H^[38]^	2020	M, 61y	US/CT/MRCP	intraoperative	OC	N
Kumar S^[39]^	2021	M, 6y	US/MRCP	intraoperative	OC	N
Singh JP^[40]^	2021	F, 60y	US/CT	intraoperative	LC	N
Wang TN^[41]^	2022	M, 63y	laparoscopy	postoperative	LC	N

EMIC = endoscopic minimally invasive cholecystolithotomy, F = female, M = male, N = no, LC = laparoscopic cholecystectomy, OC = open cholecystectomy, y = year old.

After reviewing the literature, it can be concluded that gallbladder duplication is still a rare congenital anomaly of the biliary tree. Less 50% of cases were detected via preoperative traditional imaging. Adequate preoperative evaluation is necessary for the surgeon to avoid missing gallbladder duplication. The value of using 3D reconstruction of gallbladder duplication is feasible and innovative, and facilitates guiding to LC.

## Author contributions

**Conceptualization:** Yong Qing Ye, Qing Liang.

**Data curation:** Yong Qing Ye.

**Resources:** Yong Qing Ye, En Ze Li.

**Supervision:** En Ze Li.

**Validation:** Jing Ming Fan.

**Visualization:** Yong Qing Ye, Jing Lin Gong, JingMing Fan.

**Writing – original draft:** Yong Qing Ye.

**Writing – review & editing:** Yong Qing Ye, Qing Liang, Ping Wang.
